# The complete chloroplast genome of the marine microalga *Nitzschia dubiiformis*

**DOI:** 10.1080/23802359.2022.2160672

**Published:** 2023-01-08

**Authors:** Fangfang Yang, Cheng Feng, Lijuan Long

**Affiliations:** a Key Laboratory of Tropical Marine Bio-resources and Ecology, South China Sea Institute of Oceanology, Chinese Academy of Sciences, Guangzhou, China; b University of Chinese Academy of Sciences, Beijing, PR China

**Keywords:** *Nitzschia dubiiformis*, chloroplast genome, phylogenetics

## Abstract

*Nitzschia dubiiformis* Hust 1939 is a globally distributed species belonging to the family Bacillariaceae. This study reported the complete chloroplast genome sequences of *N. dubiiformis*. The genome of *N. dubiiformis* was 179,935 bp in length, consisting of 48,722 bp of large single copy, 103,427 bp of small single copy, and 13,893 bp of a pair of inverted repeat regions. It encoded 188 genes, including 151 protein-coding genes, 6 rRNA and 31 tRNA genes. The GC content of complete chloroplast genome was 30.4%. The phylogenomic analysis suggests that there is a close relationship between *N. dubiiformis* and *N. traheaformis*.

## 1. Introduction

*Nitzschia dubiiformis* Hust 1939 belongs to genus *Nitzschia* within family Bacillariaceae (Kociolek et al. 2021). The genus *Nitzschia* has been reported firstly by Hassall in 1845, and now comprises approximately 1000 nomenclaturally valid species. They play important roles in primary production, nutrient cycling, and sediment stabilization. Several studies have revealed that *Nitzschia* sp. is a promising feedstock for biofuels and bioproducts due to strong adaptation to a wide range of environmental conditions, high growth rate and lipid content (Hildebrand et al. 2012; Anandapadmanaban et al. 2020; Oliver et al. 2021). Additionally, several strains of *Nitzschia* can produce the neurotoxin domoic acid, which can cause Amnesic Shellfish Poisoning in sea birds, mammals and humans (Kotaki et al. 2005). However, research on the genetics and evolution of *N. dubiiformis* is still extremely rare. In this study, we reported the chloroplast genome of *N. dubiiformis*, and examined its phylogenetic position within the family Bacillariaceae.

## 2. Materials

*N. dubiiformis* was originally isolated from the sea area of Sanya 18°20′ N and 109°5′ E) of Hainan Province, China. The algal cells were cultured with f/2 medium and deposited at the laboratory of the South China Sea Institute of Oceanology, Chinese Academy of Sciences, Guangzhou City, Guangdong Province (http://www.scsio.ac.cn/, Fangfang Yang, ycuyang@scsio.ac.cn) under voucher number SY20210604.

## 3. Methods

Genomic DNA was extracted using Genomic DNA Kits according to the manufacturer’s protocol. The total genomic DNA was constructed in a sequencing library with a 350 bp insert using the Nextera XT DNA library preparation kit (Illumina, San Diego, CA). Double-terminal sequencing was then performed on the library using the Illumina Novaseq 6000 sequencing platform. The raw data was edited using NGS QC Tool Kit v2.3.3 (Patel and Jain 2012). The GC content, Q20 value and Q30 value of the clean data was 49.44%, 97.47%, and 92.63%, respectively. The 4.69 G high-quality reads were assembled into chloroplast genome using a de novo assembler SPAdes v3.14.1 (Bankevich et al. 2012). Finally, the PGA program (Qu et al. 2019) was used to annotate the chloroplast genome, using the *Nitzschia traheaformis* (GenBank accession NC_061047.1) chloroplast genome as the reference. To confirm the phylogenetic position of *N. dubiiformis*, we selected the chloroplast genome sequences of 24 other species published in the NCBI to construct a phylogenetic tree. These sequences were aligned using the MAFFT version 7 software with the FFT-NS-2 strategy (Katoh and Standley 2016). The maximum likelihood tree was constructed with IQ-TREE 2.0 with 1000 bootstraps based on the maximum-likelihood method (Nguyen et al. 2015).

## 4. Results

**Figure 1.**
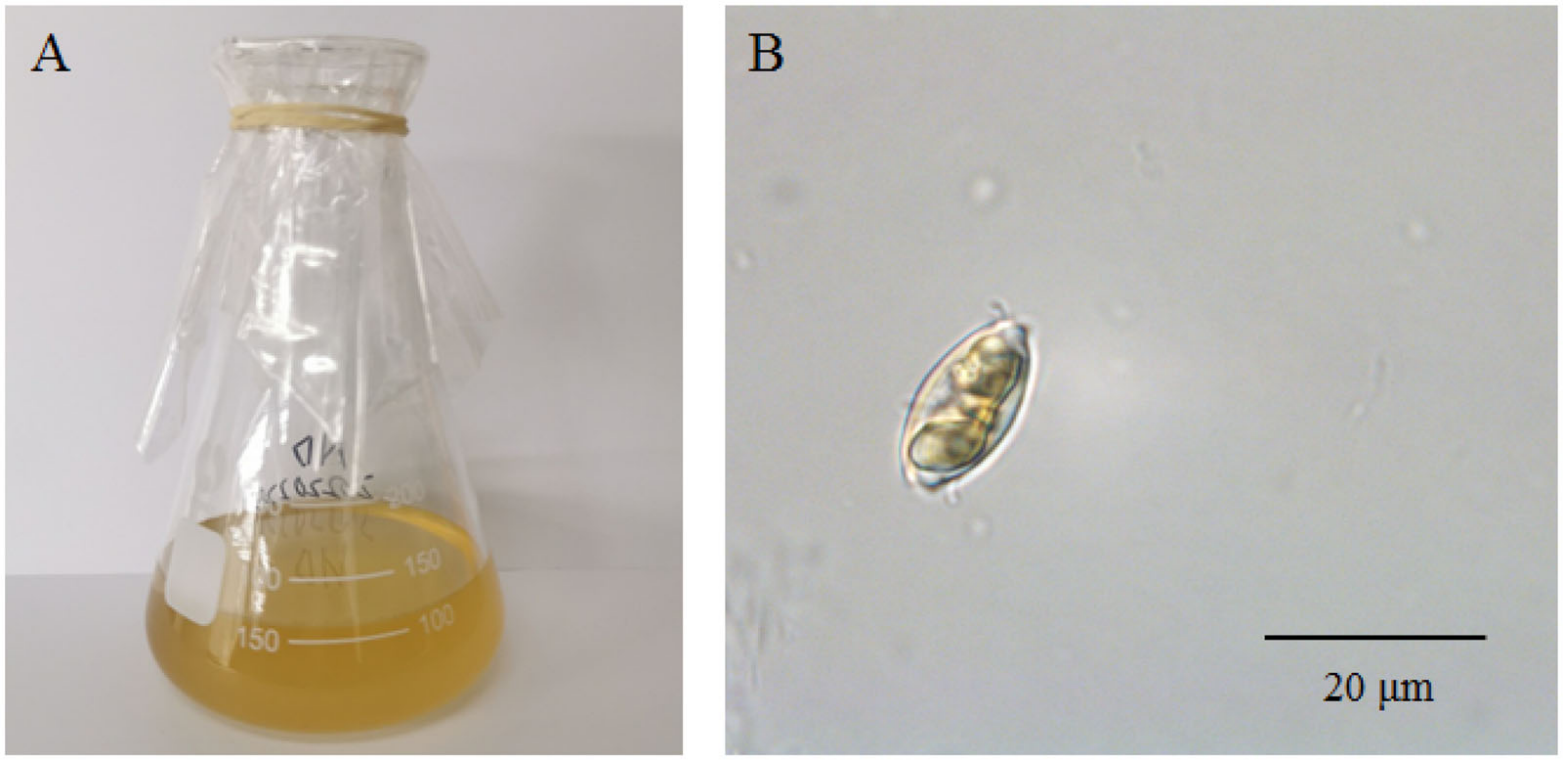
The morphology of *N. dubiiformis* (A) in flasks and (B) under a microscope. Photograph was taken by Fangfang Yang.

**Figure 2.**
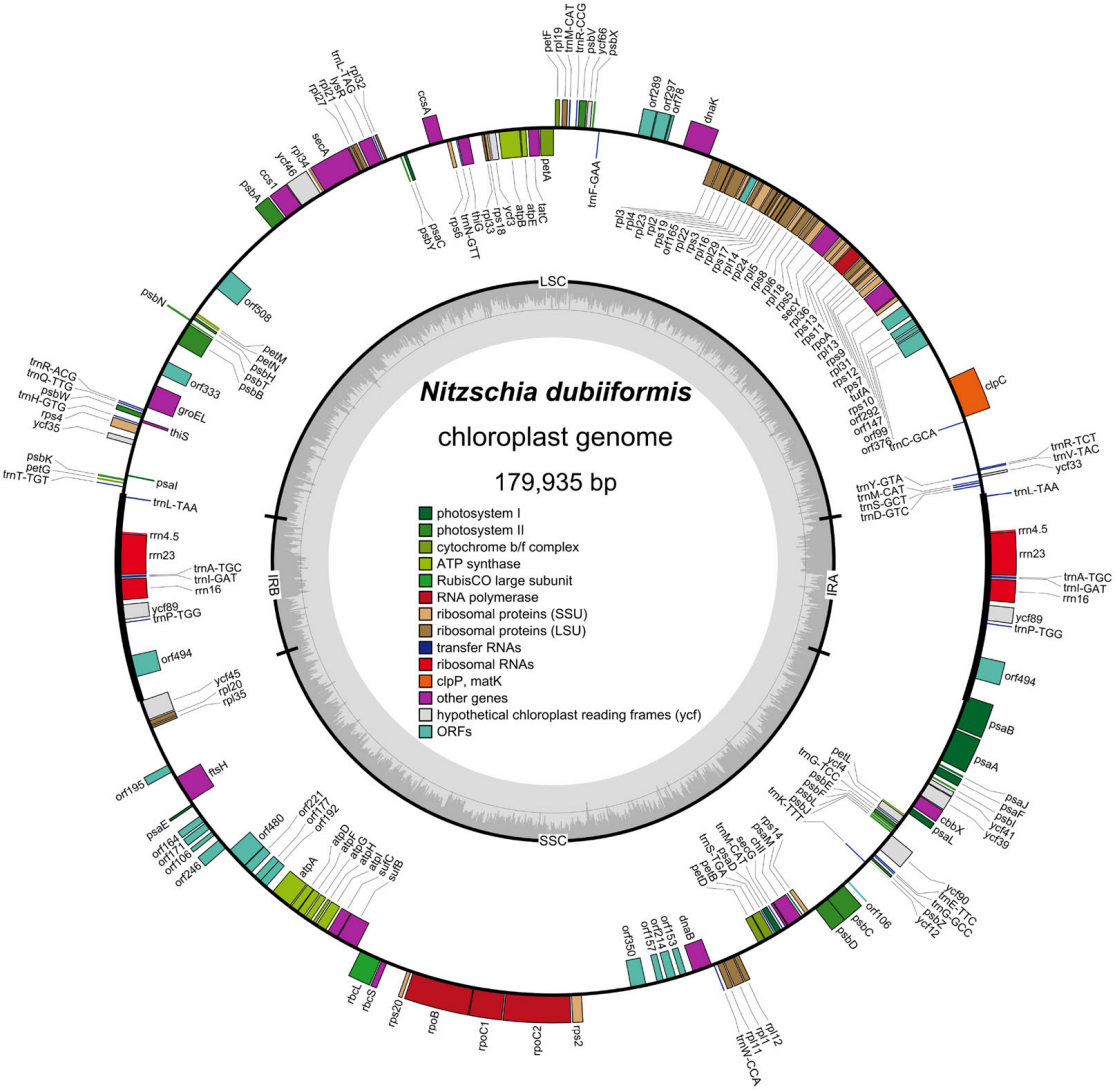
Chloroplast genome map of *N. dubiiformis*. Genes are color coded by their function in the legend. Inverted repeat (IR), small single-copy (SSC), and large single-copy (LSC) regions are indicated.

**Figure 3.**
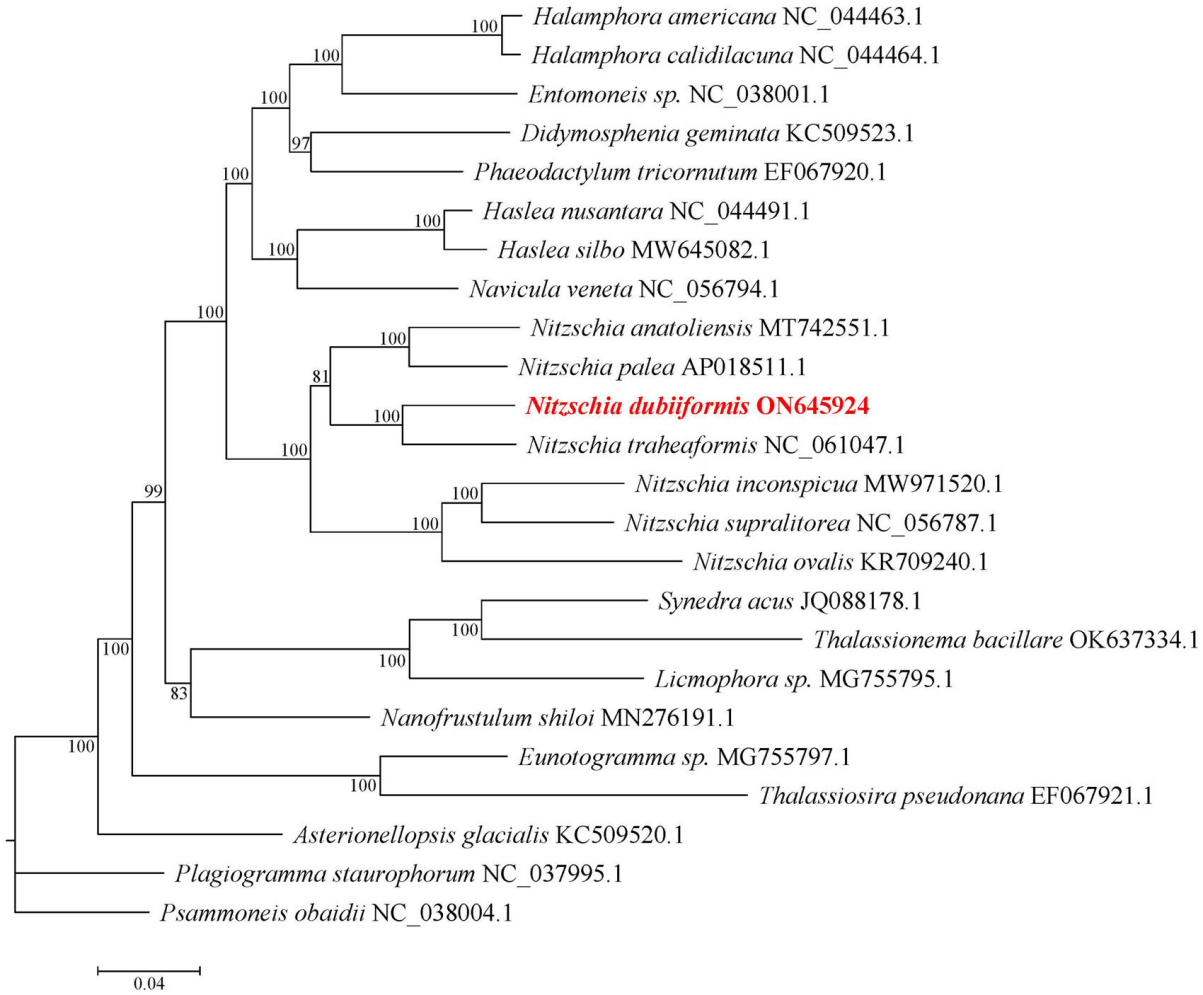
The phylogenetic tree based on the 24 complete chloroplast genome sequences. Numbers near the nodes represent maximum-likelihood bootstrap values.

The cells of *N. dubiiformis* were yellow-brown, with a visible cell wall and chloroplasts (Figure 1). The complete length of the chloroplast genome of *N. dubiiformis* (GenBank accession ON645924) presented a typical quadripartite structure with a total length of 179,935 bp (Figure 2). The large single copy region was 48,722 bp, while the small single copy region was 103,427 bp. Two inverted repeat regions were 16,158 bp. The GC content of *N. dubiiformis* genome was 30.4%. A total of 188 genes were identified, including 151 protein-coding genes, 6 rRNA and 31 tRNA genes. The phylogenetic tree supported that *N. dubiiformis* was closely related to *N. traheaformis* (Figure 3).

## 5. Discussion and conclusion

In this study, the chloroplast genome sequence of *N. dubiiformis* was first reported. These repeat motifs could be selected for developing markers and population studies. This study also provides valuable information about the evolution of the family Bacillariaceae, and improves our understanding of its taxonomic classification.

## Supplementary Material

Supplemental Material

## Data Availability

The genome sequence data that support the findings of this study are openly available in GenBank of NCBI at (https://www.ncbi.nlm.nih.gov/) under the accession no. ON645924. The associated BioProject, SRA, and Bio-Sample numbers are PRJNA845080, SRR19536226, and SAMN28854501, respectively.
