# Supplementary figures and images for: The complete chloroplast genome of the marine microalga Nitzschia dubiiformis
Source: Mitochondrial DNA B Resour. 2023 Jan 8;8(1):91–4. doi: 10.1080/23802359.2022.2160672 (PMC9833402; doi:10.1080/23802359.2022.2160672)

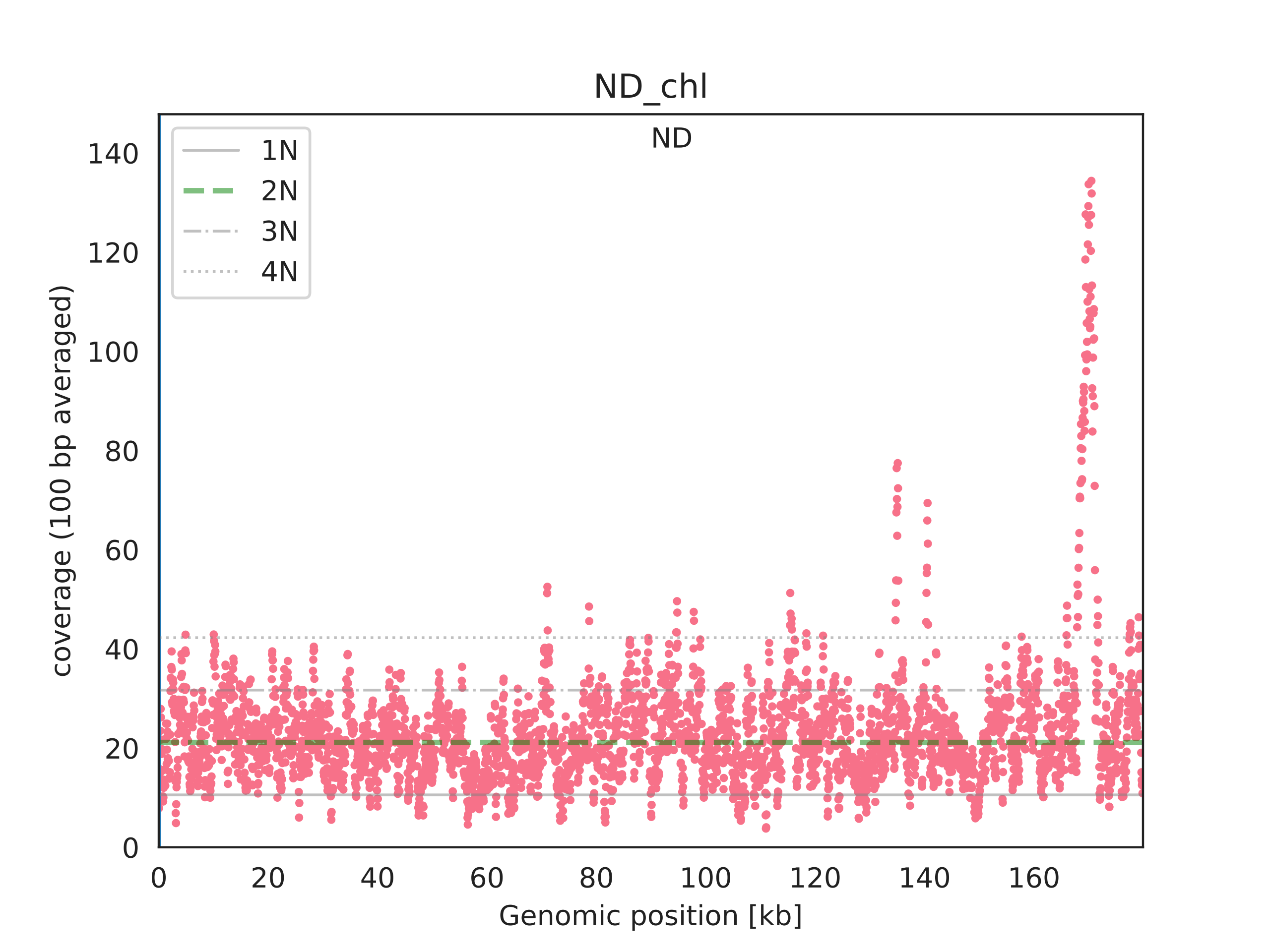


**Fig S1** The genomic position of *N. dubiiformis*.

Supplement: Supplemental Material [file TMDN_A_2160672_SM7549.doc]
